# Triptolide targets *JUN* to reverse cisplatin resistance of ovarian cancer: insights from single-cell transcriptome analysis and machine learning validation

**DOI:** 10.3389/fphar.2026.1850438

**Published:** 2026-07-09

**Authors:** Chen Wang, Junfeng Guo, Taiyang Ye, Jie Zhu, Chenhuan Ding, He Li

**Affiliations:** 1 Department of Traditional Chinese Medicine, Renji Hospital, School of Medicine, Shanghai Jiao Tong University, Shanghai, China; 2 Department of Obstetrics and Gynecology, Renji Hospital, School of Medicine, Shanghai Jiao Tong University, Shanghai, China; 3 Department of Traditional Chinese Medicine, School of Medicine, Pujiang Hospital, Minhang Campus of Renji Hospital, Shanghai Jiao Tong University, Shanghai, China

**Keywords:** ovarian cancer, Triptolide, cisplatin resistance, single-cell RNA sequencing, machine learning

## Abstract

**Background:**

Platinum-resistant ovarian cancer (PROC) is a major clinical challenge driven by profound intratumoral heterogeneity. Triptolide (TP) exhibits promising anti-tumor potential, yet its precise mechanisms within PROC remain elusive due to the limitations of traditional target-screening strategies.

**Methods:**

This study developed a comprehensive strategy integrating computational predictions with in vitro experimental validations. First, scRNA-seq data were processed to evaluate TP target genes predicted by SwissTargetPrediction, alongside pseudotime trajectory and CellChat intercellular communication analyses. Subsequently, an ensemble of six machine learning (ML) algorithms (LASSO, Random Forest, Boruta, Decision Tree, XGBoost, and GBM) was utilized to pinpoint the core therapeutic target. To verify direct molecular engagement, molecular docking, molecular dynamics (MD) simulations, and Surface Plasmon Resonance (SPR) assays were performed. Finally, the functional mechanism of the identified target in CDDP resistance was validated in vitro using parental SKOV3 and resistant SKOV3/CDDP cell lines.

**Results:**

scRNA-seq analysis revealed TP target genes are preferentially enriched in highly genomically unstable malignant epithelial cells. This subpopulation showed an aggressive intercellular communication profile, profound dependence on extracellular matrix (ECM) signals, and dominant secretion of the chemoresistance-related cytokine osteopontin (SPP1). Furthermore, the ML pipeline consistently pinpointed the proto-oncogene *JUN* as the core therapeutic target. Experiments confirmed TP efficiently suppressed aberrant c-Jun overexpression. Targeted *JUN* knockdown restored CDDP sensitivity, while its overexpression antagonized the synergistic cytotoxic and apoptotic effects of TP and CDDP. Ultimately, TP reverses CDDP resistance in PROC by downregulating *JUN*, dismantling intracellular pro-survival networks, and disrupting pro-tumorigenic crosstalk.

**Conclusion:**

In conclusion, TP reverses CDDP resistance in PROC by downregulating *JUN*, which dismantles intracellular pro-survival networks. Integrating scRNA-seq and ML provides an accurate paradigm for deciphering botanical pharmacology, laying a strong foundation for the future development of TP-based therapies tailored for PROC.

## 1 Introduction

Ovarian cancer (OC), particularly high-grade serous ovarian cancer (HGSOC), is the most lethal gynecological malignancy worldwide (Siegel et al., 2023; Matulonis et al., 2016). Despite significant advancements in therapeutic strategies centered on cytoreductive surgery and platinum-based chemotherapy, the prognosis for patients with advanced HGSOC remains dismal (Lheureux et al., 2019). Clinical data indicate that approximately 70% of patients experience a rapid therapeutic bottleneck characterized by high recurrence rates and platinum resistance following initial remission (Bowtell et al., 2015). The fundamental cause of this therapeutic dilemma is the profound heterogeneity within platinum-resistant OC (PROC) tumors, which renders conventional chemotherapies largely ineffective (Bowtell et al., 2015; Geistlinger et al., 2020). Therefore, identifying novel therapeutic strategies capable of precise intervention against malignant PROC cells is crucial to breaking the current clinical deadlock.

Among numerous natural products with anti-tumor potential, triptolide (TP), a diterpenoid triepoxide lactone derived from the traditional Chinese herb *Tripterygium wilfordii Hook F.,* has garnered significant attention due to its unique capacity for multi-target synergy and robust inhibition of tumor progression (Tong et al., 2021; Titov et al., 2011). Although substantial evidence confirms that TP can inhibit proliferation, induce apoptosis, and block metastasis across various solid tumors (Noel et al., 2019; Li et al., 2014), its precise mechanisms of action within the highly heterogeneous context of PROC remain elusive (Zhong et al., 2013).

Existing mechanistic studies predominantly rely on bulk transcriptomics, an approach that typically treats tumor tissue as a homogeneous entity. This method inherently obscures the drug response characteristics of low-abundance resistant cells and distinct cellular subpopulations (Lim et al., 2020; Lawson et al., 2018). Currently, it remains unclear exactly how TP targets tumor cells. It is also not fully understood whether TP remodels the tumor microenvironment (TME) to exert its effects (Meng et al., 2014). These knowledge gaps significantly hinder the clinical translation of TP (Gao et al., 2021).

To overcome these methodological limitations, we propose a novel target-screening strategy that integrates single-cell RNA sequencing (scRNA-seq) data with machine learning (ML) algorithms (Suva and Tirosh, 2019). Specifically, mining scRNA-seq data allows for the precise deconstruction of the intricate intratumoral cellular landscape, enabling the identification of key cellular subpopulations that are highly sensitive to TP (Stuart and Satija, 2019). Furthermore, applying ML algorithms to these high-dimensional datasets facilitates dimensionality reduction and feature extraction, thereby efficiently pinpointing essential feature genes (Eraslan et al., 2019; Luecken and Theis, 2019).

Ultimately, this study aims to employ this integrated strategy to precisely identify the pharmacological targets of TP and validate them experimentally. These findings will provide a robust theoretical foundation for the development of TP-based therapeutic regimens for OC platinum resistance.

## 2 Methods

### 2.1 Data acquisition

A total of 12 scRNA-seq samples, including 7 OC without treatment and 5 normal ovary samples, was procured from the GEO database (https://www.ncbi.nlm.nih.gov/geo/) under accession ID: GSE184880.

### 2.2 Prediction of potential drug targets

To identify potential biological targets of TP, we utilized the SwissTargetPrediction web server (http://www.swisstargetprediction.ch/). The canonical SMILES string of TP was obtained from the PubChem database and submitted to the server. The prediction algorithm combines 2D chemical similarity and 3D molecular shape comparisons to match the query molecule against a curated collection of known actives. The species was limited to “*Homo sapiens*”. Targets were ranked based on their probability scores, which represent the likelihood of the compound binding to a specific protein.

### 2.3 Construction of protein-protein interaction (PPI) network

To explore the functional connectivity and potential mechanism of TP, the candidate target genes identified via SwissTargetPrediction were imported into the GeneMANIA database (http://genemania.org/). The organism was set to “*Homo sapiens*”. A composite gene interaction network was constructed based on multiple genomic and proteomic data sources, including physical interactions, co-expression, co-localization, genetic interactions, and pathway associations. Genes with similar functions to the query list were prioritized, and the resulting network was visualized to elucidate the synergistic relationships among the potential targets of TP.

### 2.4 Functional enrichment analysis

GO annotation and KEGG pathway enrichment analysis were conducted using the “clusterProfiler” package in R software. The analysis covered biological processes (BP), cellular components (CC), and molecular functions (MF). The *p* values were adjusted using the Benjamini-Hochberg method to control the false discovery rate. Terms and pathways with an adjusted *p* < 0.05 were considered statistically significant and visualized.

### 2.5 scRNA-seq data processing and analysis

The scRNA-seq data were processed and analyzed using the “Seurat” R package. Cells expressing fewer than 200 genes were excluded to filter out empty droplets and poor-quality cellular debris. Conversely, an upper limit of 7,000 genes was applied to rigorously eliminate potential doublets or multiplets, ensuring the reliability of the downstream single-cell resolution. HGSOC is highly heterogeneous, metabolically active, and prone to hypoxia. Based on the overall distribution of the dataset and standard practices for aggressive solid tumors, a 20% threshold was selected to retain sufficient biologically viable malignant epithelial cells while removing undeniably dying or apoptotic cells. The filtered single-cell expression profile was first subjected to log-normalization and linear regression scaling. Utilizing the FindVariableFeatures algorithm within Seurat, we identified the top 2,000 highly variable genes (HVGs) for downstream Principal Component Analysis (PCA). To align datasets and eliminate confounding batch effects across multi-sample cohorts, the Harmony framework was employed for data integration. For cellular programmatic characterization, low-dimensional embedding and cell clustering were projected via t-Distributed Stochastic Neighbor Embedding (t-SNE). Main cell lineages were assigned and validated using a canonical marker-based annotation approach. To further scrutinize target-driven phenotypic shifts, we performed focused unsupervised re-clustering specifically within the Epithelial domain, stratifying epithelial subpopulations based on their granular computational profiles while avoiding over-parameterization.

Cell types were annotated based on three criteria: significantly elevated expression of marker genes, unique gene expression patterns, and alignment with established canonical markers from the literature. Differential gene expression analysis to identify these marker genes was performed using the default Wilcoxon rank-sum test in the *Seurat* package, and p-values were adjusted for multiple testing utilizing the Bonferroni correction method.

### 2.6 Evaluation of SwissTarget Score in epithelial cells

To evaluate the aggregate expression potential of TP targets within the epithelial cell population, a gene signature was constructed using the candidate target genes identified by SwissTargetPrediction. A composite expression score, termed the “SwissTarget Score”, was calculated for each individual cell using the AddModuleScore function in the “*Seurat”* R package. Based on the distribution of these scores, cells were stratified into three groups using quartile thresholds: Low (< 25th percentile), Medium (25th - 75th percentile), and High (> 75th percentile). Furthermore, to computationally infer malignant profiles within the extracted epithelial cell compartment, single-cell chromosomal copy number variant (CNV) analysis was executed using the “*CopyKAT*” R package. *CopyKAT* implemented an unsupervised Bayesian approach integrated with hierarchical clustering to establish single-cell genomic copy number matrices. To smooth the gene expression signals and ensure statistical robustness, the algorithm’s default parameter settings were strictly applied, including a sliding window of 25 genes (win.size = 25) and a minimum constraint of 5 genes required per chromosome (ngene.chr = 5). Cells were partitioned based on the default clustering configurations into three definitive structural states: Normal, not.defined, and Tumor. Crucially, this inferred CNV stratification was utilized as a genomic validation baseline to support malignant epithelial cell-state annotations. To further validate whether genomic changes driven transcription alterations, CNV-expression correlation analysis (cnv_expr_cor) was performed between regional genomic copy profiles and locus-specific transcription levels, accompanied by comparative statistics for customized key feature genes. Additionally, the continuous SwissTarget module scores were quantitatively mapped across these states via Spearman rank correlation (ρ), and the difference in SwissTarget scores between the defined Tumor and Normal clusters was statistically assessed using the Wilcoxon rank-sum test.

### 2.7 Pseudotime trajectory analysis

To characterize the differentiation states and evolutionary lineage of epithelial cells, single-cell trajectory analysis was performed using the “*Monocle*” R package. The top 2,000 highly variable genes (HVGs) were selected as ordering genes to construct the trajectory. Dimensionality reduction and trajectory construction were conducted using the DDRTree algorithm. To maintain consistent visual and spatial alignment with the global cell clustering map, the resulting multi-branch trajectory tree topology was projected and visualized within the 2D t-SNE embedding space. To determine the biological directionality of cell-state progression, CytoTRACE-high cells, which represent the least differentiated, stem-like, and most primitive cellular states, were designated as the default trajectory root (Pseudotime=0). This configuration established a developmental baseline to track continuous pseudo-temporal transcriptomic shifts and transitions toward the malignant OC phenotype. Following this, we investigated the dynamic changes of the TP target signature along this trajectory. Pseudotime distributions were statistically compared across the distinct SwissTarget score groups (Low, Medium and High), and the correlation between the SwissTarget Score and pseudotime was analyzed in both normal and tumor contexts to elucidate the relationship between target expression and cellular differentiation status.

### 2.8 Cell-cell interaction analysis

To elucidate the cellular interactome and characterize potential molecular cross-talk driven by TP target heterogeneity, cell-cell communication analysis was performed using the “*CellChat*” R package. Crucially, to establish a comprehensive comparative analytical framework, epithelial cells were stratified into three distinct sub-populations based on their SwissTargetPrediction module score quartiles. Single-cell gene expression matrices paired with both global cell-type annotations and these stratified epithelial sub-populations were imported to construct corresponding CellChat objects. The database of human ligand-receptor interactions embedded within CellChat was utilized as the reference template. Intercellular communication probability and signaling network intensities were quantitatively modeled using the statistical triMean approximation method. To eliminate artifacts arising from underrepresented populations and ensure statistical reliability, cell groups containing fewer than 10 cells were strictly excluded from the communication network projection framework (minimum cells per group = 10). Differential signaling flow, pathway network weights, and specific ligand-receptor interactions were subsequently compared across the Epithelial_High, Epithelial_Medium, and Epithelial_Low sub-populations to dissect the impact of target expression heterogeneity on the tumor interactome.

### 2.9 Identification of TP target genes in OC

To identify feature genes critically involved in TP regulation, we integrated six ML algorithms: Least Absolute Shrinkage and Selection Operator (LASSO), Random Forest (RF), Boruta, Decision Tree (DT), eXtreme Gradient Boosting (XGBoost), and Gradient Boosting Machine (GBM). All analyses were performed in R (version 4.1.3). The single-cell dataset was randomly stratified into a training set (70%) and an internal testing set (30%). To prevent class-imbalance-induced overfitting, the input dataset was strictly balanced, utilizing an equal number of cells for the positive (High-score, n = 814) and negative (Low-score, n = 814) classes. To optimize predictive precision and further control overfitting, six independent algorithms were executed with specific hyperparameter configurations and a globally fixed random seed (12345) to ensure exact numerical reproducibility. Specifically, LASSO logistic regression was optimized via 10-fold cross-validation to pinpoint the optimal penalty threshold; RF was configured with 500 independent decision trees; Boruta was run iteratively with a maximum cutoff of 100 runs; DT applied a recursive splitting complexity parameter of 0.001; GBM utilized 500 trees, an interaction depth of 3, and a 0.01 shrinkage learning rate; and XGBoost was constrained to 100 boosting rounds, a maximum tree depth of 3, and a 0.1 learning step contraction rate (η). To mitigate individual structural biases and prevent overfitting, a multi-method voting strategy was applied, wherein a candidate gene was retained as a definitive feature only if concurrently selected by ≥ 4 out of the 6 computational methods.

### 2.10 Diagnostic relevance of optimal feature genes

To evaluate the predictive performance of candidate genes, Receiver Operating Characteristic (ROC) curve analysis was performed. The ROC curves were generated by plotting the True Positive Rate (Sensitivity) against the False Positive Rate (1-Specificity) across various threshold settings. The Area Under the Curve (AUC) was calculated to quantify the overall discriminative ability of each biomarker, with an AUC value closer to 1.0 indicating superior predictive performance. For each gene, the optimal cut-off value was determined using the Youden index, which balances sensitivity and specificity to maximize the correct classification rate. Based on this optimal threshold, samples were categorized into predicted high and predicted low groups, and confusion matrices were constructed to visualize the classification performance. Crucially, this analytical pipeline was explicitly designed for exploratory, cell-level candidate gene prioritization. Acknowledging the lack of independent external validation cohorts, this approach serves as a descriptive methodology rather than the construction of a validated, clinically deployable diagnostic prediction model.

### 2.11 Survival analysis using public databases

The prognostic value of the *JUN* gene in OC was evaluated using the Kaplan-Meier Plotter database (http://kmplot.com/analysis/). Patient samples were split into high and low expression groups by the median expression of the probe (201466_s_at). Overall survival (OS) and progression-free survival (PFS) curves were generated, and the Hazard Ratio (HR) with 95% confidence intervals and log-rank p-values were calculated automatically by the database tool.

### 2.12 Molecular docking

Molecular docking was employed to explore the binding mode between TP and the core target protein c-Jun. The SDF structure file of TP was downloaded from the PubChem database (Compound CID:107985), and the high-resolution PDB file of the c-Jun protein was obtained from the RCSB PDB database (PDB ID: 6Y3V). Both TP and c-jun protein structure files were prepared and processed via AutoDock software (version 1.5.6). AutoDock Vina was then run to dock the processed ligand to the target protein for 10 independent runs, and the conformation with the lowest binding energy from these runs was utilized as the final optimal result. Finally, the docking results were visualized and analyzed using PyMOL software, with a specific focus on characterizing the non-covalent interactions, particularly the stable hydrogen bonding network formed between the ligand and key amino acid residues within the active pocket of c-Jun.

### 2.13 Molecular dynamics simulation

Molecular dynamics (MD) simulations of the protein-ligand complex were performed using GROMACS (version 2022.5) software. The protein topology was generated employing the AMBER99SB-ILDN force field, while the ligand topology was constructed using the Amber force field via the ACPYPE script. The docking complex was solvated in a cubic box filled with TIP3P water molecules under periodic boundary conditions. To maintain system neutrality, NaCl counterions were appropriately added.

Prior to the MD simulations, energy minimization was executed utilizing the steepest descent algorithm to remove unfavorable steric clashes, with both the Coulomb and van der Waals cutoff radii set to 1.4 nm. Subsequently, the system was equilibrated under NVT (constant number of particles, volume, and temperature) and NPT (constant number of particles, pressure, and temperature) ensembles for 100 ps each to achieve stable temperature and pressure. The LINCS algorithm was applied to constrain all covalent bonds, and long-range electrostatic interactions were computed using the Particle Mesh Ewald (PME) method. Finally, a 100 ns production MD simulation was conducted under periodic boundary conditions at a physiological temperature of 310 K and a constant pressure of 1.0 bar.

Following the simulation, trajectory analyses were systematically performed. The Root Mean Square Deviation (RMSD) of the backbone atoms was calculated to assess the conformational stability and structural equilibrium of the complex relative to its initial state. Root Mean Square Fluctuation (RMSF) was utilized to evaluate the local flexibility and atomic positional fluctuations of individual residues during the simulation. Additionally, the number of intermolecular hydrogen bonds, a critical parameter for evaluating binding affinity, was dynamically monitored throughout the trajectory.

### 2.14 Surface Plasmon Resonance (SPR) assay

The binding affinity between the c-Jun protein and TP was determined using SPR biosensor system, Biacore 8K (Cytiva, USA). A standard CM5 sensor chip was employed for the assay. The chip surface was first activated with a freshly prepared 1:1 mixture of 400 mM N-ethyl-N'-(3-dimethylaminopropyl) carbodiimide (EDC) and 100 mM N-hydroxysuccinimide (NHS) for 420 s at a flow rate of 10 μL/min. Subsequently, the purified JUN protein, diluted to 20 μg/mL in 10 mM sodium acetate buffer (pH 4.5), was injected into the sample channel (Fc2) to achieve an immobilization level of approximately 12,600 response units (RU). The reference channel (Fc1) was left unmodified. Unreacted active ester groups on the chip surface were then blocked by injecting 1 M ethanolamine-HCl (pH 8.5) for 420 s at 10 μL/min.

For kinetic multi-cycle analysis, TP was serially diluted in the running buffer (1×PBS containing 1% DMSO and 0.005% Tween 20) to generate a concentration gradient (1.56, 3.13, 6.25, 13.00, 25.00, 50.00, and 100.00 μM). Each concentration of TP was injected over both Fc1 and Fc2 at a constant flow rate of 20 μL/min, with an association phase of 100 s followed by a dissociation phase of 180 s. After each cycle, the sensor chip was regenerated using 10 mM Glycine-HCl (pH 2.0) to restore baseline conditions. All binding data were processed and fitted to a 1:1 Langmuir binding model utilizing the Biacore Insight Evaluation software to calculate the equilibrium dissociation constant (KD).

### 2.15 Cell culture

The human OC cell line SKOV3, and CDDP-resistant human OC cell line SKOV3/CDDP were provided by the State Key Laboratory of Oncogenes and Related Genes (Shanghai, China). All cells were cultured in DMEM containing 10% FBS and 1% penicillin-streptomycin at 37 °C with 5% (v/v) CO_2_.

### 2.16 Materials and reagents

TP (purity=99.94%) and c-jun protein were obtained from MedChemExpress (USA). Compound purity and structural validation of TP were shown in Supplementary Figures S3, S4. Trypsin-EDTA (0.25%), DMEM, FBS, and penicillin-streptomycin were obtained from Gibco (USA). Primary antibodies against c-Jun (#9165, rabbit) and GAPDH (#97166, mouse) were purchased from Cell Signaling Technology (USA).

### 2.17 Quantitative real-time PCR (qRT-PCR)

Total RNA was extracted using TRIzol Reagent (Invitrogen, USA). Subsequently, reverse transcription into complementary DNA was performed utilizing the PrimeScript™ RT Reagent Kit (Takara, Japan) according to the manufacturer’s instructions. The relative mRNA expression levels were quantified via qRT-PCR with SYBR Green and calculated using the 2^−ΔΔCT^ method. The gene-specific primers were synthesized by Sangon (Shanghai, China), and their sequences were as follows: *JUN*, 5’-CCT​TGA​AAG​CTC​AGA​ACT​CGG​AG-3’ (forward) and 5’-TGC​TGC​GTT​AGC​ATG​AGT​TGG​C-3’ (reverse); *GAPDH*, 5’-TGC​ACC​ACC​AAC​TGC​TTA​GC-3’ (forward) and 5’-GGC​ATG​GAC​TGT​GGT​CAT​GAG-3’ (reverse).

### 2.18 Western blot analysis

Western blot was performed as previously described (Ding et al., 2025). Briefly, following the designated treatments, total protein was extracted from SKOV3/CDDP cells using RIPA lysis buffer supplemented with protease and phosphatase inhibitors. Protein concentrations were then determined using a BCA protein assay. Equal amounts of protein samples were resolved by SDS-PAGE and subsequently transferred to PVDF membranes. After blocking with 5% non-fat milk in TBST, the membranes were probed overnight at 4 °C with primary antibodies against c-Jun and GAPDH. Following standard washing steps, the membranes were incubated with the corresponding HRP-conjugated secondary antibodies for 1 h at room temperature. Finally, the bands were visualized using a Bio-Rad Imaging System.

### 2.19 Small interfering RNA (siRNA) transfection

siRNAs specifically targeting the human *JUN* gene (si-JUN), alongside a non-targeting negative control siRNA (si-NC), were synthesized by GenePharma. Transfection of the siRNA duplexes was performed using Lipofectamine 2000 reagent (Invitrogen, USA) following the manufacturer’s instructions. 24 h after transfection, the mRNA expression levels of the target gene were evaluated via RT-qPCR, with melt curve analysis conducted to confirm primer specificity. Subsequently, target protein levels were assessed by western blot 48 h after transfection.

### 2.20 CCK-8 assay

CCK-8 assays were performed to determine IC_50_ values and cell viability. Initially, the respective cells were seeded into 96-well plates at a density of 5×10^3^ cells per well and incubated overnight. For the determination of IC_50_ values, SKOV3, SKOV3/CDDP and SKOV3/CDDP cells transfected with si-NC or si-JUN were treated with various concentrations of CDDP for 48 h. For the cell viability assay, SKOV3/CDDP, SKOV3/CDDP-JUN-EV, and SKOV3/CDDP-JUN-OE cells were exposed to different drugs for 48 h. Following the treatments, 100 µl of DMEM containing 10% CCK-8 solution (DOJINDO, Japan) was added to each well, and the plates were further incubated for 1 h at 37 °C. Finally, the absorbance values were measured at 450 nm using a microplate spectrophotometer.

### 2.21 Establishment of *JUN*-overexpressing stable cell lines

GFP-tagged lentiviral vectors overexpressing *JUN* (Lv-JUN-OE) and empty control vectors (Lv-JUN-EV) were obtained from GenePharma (Shanghai, China). SKOV3/CDDP cells were transfected with the lentiviruses in the presence of 5 μg/mL polybrene. 72h after transfection, positively infected cells were selected using culture medium containing 2 μg/mL puromycin, until mock-infected cells completely died. Puromycin-resistant cells were then isolated into single cell clones using the limiting dilution method in 96-well plates. Following monoclonal expansion, the overexpression efficiency of *JUN* in the selected clones was verified using RT-qPCR and western blot. The clone with the highest target gene expression was selected for subsequent assays.

### 2.22 Apoptosis analysis

SKOV3/CDDP, SKOV3/CDDP-JUN-EV, and SKOV3/CDDP-JUN-OE cells were seeded into 6-well plates at a density of 2×10^5^ cells/well and incubated overnight. Subsequently, the cells were treated with CDDP in the presence or absence of TP for 48h. Cell apoptosis was evaluated using an Annexin V-FITC/PI double staining kit (BD Biosciences, USA) according to the manufacturer’s instructions. Briefly, the cells were harvested, washed three times, and resuspended in 200 µL binding buffer. The cell suspensions were then stained with 5 µL of Annexin V-FITC and 5 µL of PI for 15 min at room temperature in the dark. Finally, the apoptosis rate was analyzed via flow cytometry.

### 2.23 Statistical analysis

Statistical analysis and visualization were executed in R (version 4.1.3). Statistical analyses were conducted according to the data type and experimental design. To address multiple testing in high-dimensional datasets, p-values were strictly adjusted: the Benjamini-Hochberg method was applied for functional enrichment analyses, and the Bonferroni correction was utilized for single-cell DEG analysis. For *in vitro* experiments, the two-sided Student's t-test was used for comparisons between two groups. ANOVA was utilized for experiments involving three or more groups. In all analyses, statistical significance was defined as p < 0.05.

### 2.24 Software and R package version

All computational analyses were performed using R software (version 4.1.3). The following R packages were used with their respective versions: *Seurat* (version 4.3.0), *Harmony* (version 0.1.1), *CopyKAT* (version 1.1.0), *Monocle* (version 2.22.0), *CellChat* (version 1.6.1), *clusterProfiler* (version 4.4.4), *glmnet* (version 4.1-4), *randomForest* (version 4.7-1.1), *Boruta* (version 8.0.0), *rpart* (4.1.24), *xgboost* (version 1.7.3.), *gbm* (version 2.1.8) and *ggplot2* (version 3.4.0).

## 3 Results

### 3.1 Identification of TP target genes

**FIGURE 1:**
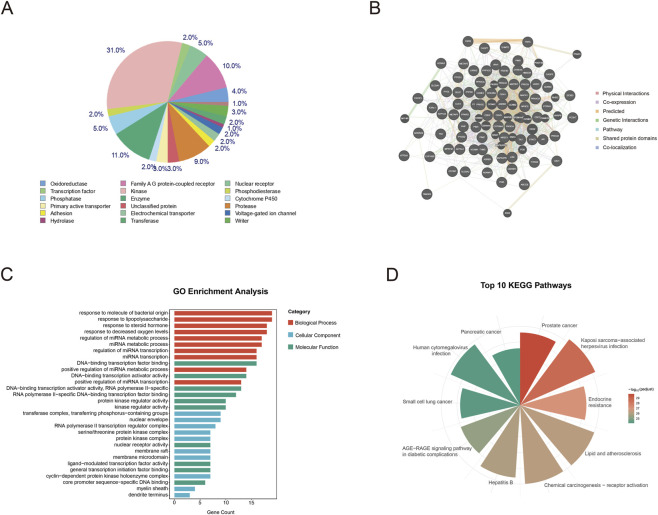
Prediction and functional characterization of candidate targets for TP. **(A)** Classification of the candidate targets based on their functions. A pie chart displays the distribution of identified candidate targets across various protein families. Prominent classes include Oxidoreductase, Transcription factor, Phosphatase, Primary active transporte, Adhesion, Hydrolase, Family A G protein-coupled receptor, Kinase, Enzyme, Unclassified protein, Electrochemical transporter, Transferase, Nuclear receptor, Phosphodiesterase, Cytochrome P450, Protease, Voltage-gated ion channel, and Writer. **(B)** Protein-protein interaction (PPI) network constructed using GeneMANIA. The network connects the identified candidate targets based on physical interactions, co-expression, predicted relationships, genetic interactions, shared pathways, and other factors. **(C)** GO enrichment analysis of the candidate targets. The bar chart shows the most significantly enriched GO terms across three categories: Biological Process (BP), Cellular Component (CC), and Molecular Function (MF). **(D)** KEGG pathway enrichment analysis of the candidate targets. The circular plot visualizes the top 10 most significantly enriched KEGG pathways. The size of each segment corresponds to the number of genes in the pathway, and the color gradient indicates the statistical significance of the enrichment, with red indicating high significance and green indicating relatively lower significance.

To elucidate the functional mechanisms of TP, 100 potential targets were identified using SwissTargetPrediction (Supplementary Table S1). Figure 1A illustrates the diverse functional classification of these targets, with the largest proportion being Kinase (31.0%). The PPI network (Figure 1B) revealed a complex interactome, suggesting that these targets operate synergistically, primarily through co-expression and physical interactions-rather than in isolation. GO analysis (Figure 1C) further characterized these targets, showing significant enrichment in stress and immune-related biological processes. Molecularly, the targets function largely in transcription factor binding and kinase regulation. Furthermore, these targets are predominantly localized to the nuclear envelope and membrane rafts. Finally, KEGG pathway analysis (Figure 1D) implicated these targets in multiple disease pathways, including prostate cancer, lipid atherosclerosis, and endocrine resistance. Notably, the AGE-RAGE signaling pathway and various viral infection pathways were also enriched, underscoring a potential multi-pathway regulatory mechanism of TP.

### 3.2 scRNA-seq analysis revealed cell profiles in OC

**FIGURE 2:**
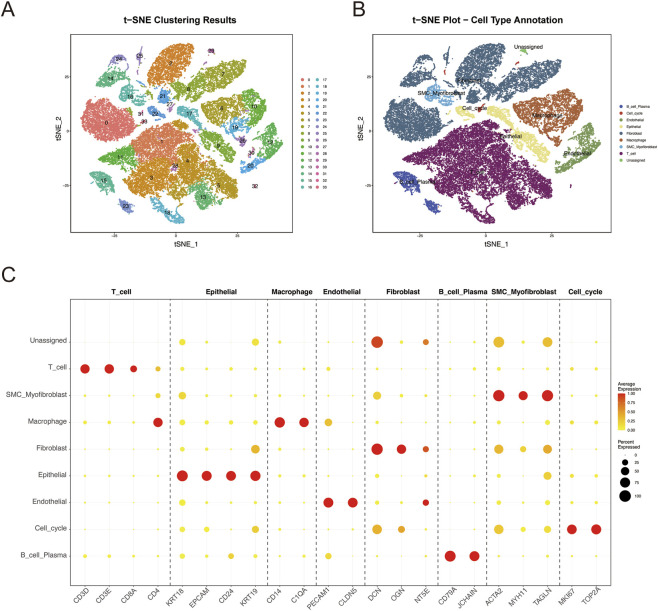
Single-cell transcriptomic landscape of OC. **(A)** The t-SNE visualization of single cells clustered based on global gene expression profiles reveals the extensive transcriptomic heterogeneity within the tumor microenvironment. The analysis partitioned the cells into 34 distinct unsupervised clusters (numbered 0 to 33), as indicated by the different colors. **(B)** Annotation of major cell types projected onto the t-SNE map. The unsupervised clusters were assigned to specific cellular lineages based on established marker genes, identifying eight major populations: T cells, Epithelial cells, Macrophages, Endothelial cells, Fibroblasts, B cells/Plasma cells, Smooth Muscle Cells (SMC)/Myofibroblasts, and a highly proliferative Cell_cycle group. A small subset of cells lacking clear lineage signatures was labeled as Unassigned. **(C)** Dot plot validating the cell type annotations by illustrating the expression patterns of canonical marker genes across the identified cell types. The x-axis groups the key lineage-specific markers utilized for annotation. The dot size indicates the percentage of cells within a specific cell type expressing a given marker, and the color gradient represents the scaled average expression level. The plot demonstrates the high specificity and robust expression of these canonical markers in their corresponding annotated cell populations.

To investigate the cellular heterogeneity within the tissue microenvironment, we performed unsupervised clustering analysis on the quality-controlled scRNA-seq data (Supplementary Figure S1). Data integration using the “harmony” algorithm effectively mitigated batch effects, resulting in a homogeneous cellular distribution across samples. This confirms the suitability of the dataset for downstream analyses. Using t-SNE for dimensionality reduction, we identified 33 distinct cell clusters based on their transcriptomic profiles (Figure 2A). We further annotated these clusters into 8 major cell lineages based on the expression of canonical marker genes (Figure 2B). The specific expression patterns of these markers are visualized in the dot plot (Figure 2C). Immune cell populations were clearly distinguished, including T cells, Macrophages, and B cells. Notably, the Macrophage cluster exhibited endogenous expression of CD4, consistent with established physiological profiles of human tissue macrophages (Dalgleish et al., 1984; Crocker et al., 1987; Cheng et al., 2021), while being firmly negative for pan-T cell markers like CD3D and CD3E. Stromal and structural components were also comprised of distinct populations. Epithelial cells were identified by the robust expression of *KRT18*, *EPCAM*, *CD24*, and *KRT19*. Endothelial cells specifically expressed *PECAM1* and *CLDN5*. Fibroblasts were characterized by the expression of *DCN*, *OGN*, and *NT5E*, while smooth muscle cells/myofibroblasts showed high expression levels of *ACTA2*, *MYH11*, and *TAGLN*. Additionally, a proliferating cell population (Cell_cycle) was identified based on the expression of cell cycle-related genes such as *MKI67* and *TOP2A*.

Collectively, this analysis provides a high-resolution map of the cellular composition, revealing a complex ecosystem composed of diverse immune, epithelial, and stromal cell subsets.

### 3.3 Characterization of TP target gene signatures in OC epithelial cells

**FIGURE 3:**
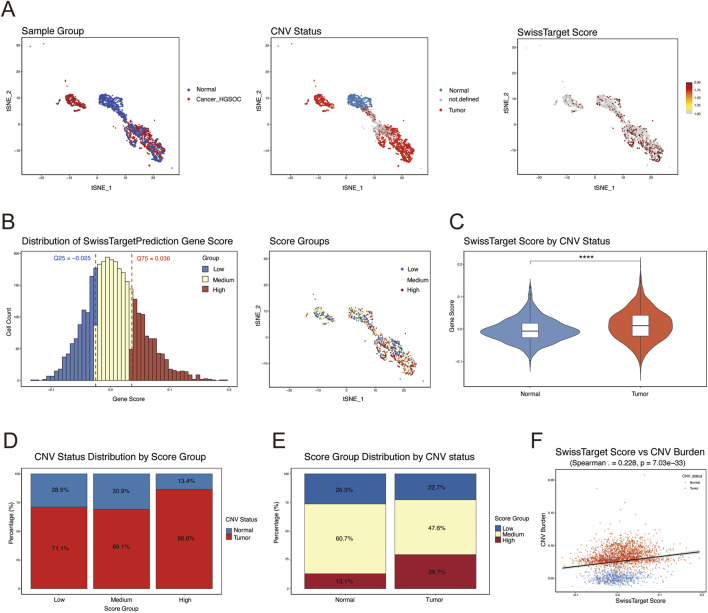
Association of the predicted TP target gene signature with tumor malignancy in single-cell transcriptomics. **(A)** t-SNE visualizations of single cells demonstrating the distribution of sample origin (left), inferred CNV status (middle), and the evaluated SwissTargetPrediction score for TP targets (right). Visual comparison reveals that cells with high SwissTarget score largely co-localize with the tumor cell clusters characterized by aberrant CNV. **(B)** Stratification of cells based on the SwissTarget score. The left histogram shows the distribution of gene scores, dividing cells into Low (< Q25 = −0.025), Medium, and High (> Q75 = 0.036) score groups. The right t-SNE plot projects these defined groups onto the cellular landscape, highlighting the distinct localization of the High-score group within the malignant population. **(C)** Violin plot comparing the SwissTarget scores between normal and tumor cells. Tumor cells exhibit a significantly elevated gene expression score for predicted TP targets compared to normal cells. **(D,E)** Stacked bar charts detailing the compositional differences across groups. Panel **(D)** shows the proportion of normal and tumor cells within each SwissTarget score group, demonstrating that the High-score group is predominantly composed of tumor cells (86.6%). Conversely, panel **(E)** displays the distribution of SwissTarget score groups within normal and tumor samples, revealing a notably higher fraction of High-score cells in the tumor group (29.7%) compared to the normal group (13.1%). **(F)** Scatter plot with Spearman correlation analysis revealing a modest positive correlation between the SwissTarget score and overall CNV burden (R = 0.228, p = 7.03e-33). ****, p < 0.0001.

Given that malignant epithelial cells play a central role in the development and progression of OC, directly determining drug resistance, this study initially focused on this subpopulation to comprehensively evaluate the potential sensitivity of OC epithelial cells to TP. We mapped the target gene set retrieved from SwissTargetPrediction onto the OC single-cell dataset. The epithelial cells were visualized using t-SNE, revealing distinct clusters for normal and tumor samples (Figure 3A). Based on the distribution of SwissTargetPrediction gene scores, cells were stratified into Low, Medium, and High groups using the 25th (Q25 = −0.025) and 75th (Q75 = 0.036) percentiles as cutoffs (Figure 3B). Quantitative analysis demonstrated that the SwissTarget scores were significantly elevated in tumor cells compared to normal epithelial cells (Figure 3C), suggesting a higher potential responsiveness of malignant cells to TP. Consistently, the High-score group was predominantly composed of tumor cells (86.6%), whereas the proportion of tumor cells was lower in the Medium (69.1%) and Low (71.1%) score groups (Figure 3D). Conversely, a larger fraction of tumor cells fell into the High-score category compared to normal cells (Figure 3E). Furthermore, we observed a modest but statistically significant positive correlation between the SwissTarget score and CNV burden (Spearman R = 0.228, p < 0.001, Figure 3F). Rather than serving as a sole determinant, this trend suggests that genomic instability acts as one of several contributing factors associated with TP target gene expression within the highly heterogeneous tumor microenvironment. Furthermore, considering that the inherent sparsity and frequent dropout events in scRNA-seq data fundamentally compress correlation magnitudes (Gan et al., 2022), this association remains biologically meaningful.

In summary, these data indicate that TP target genes are preferentially enriched in OC tumor cells, particularly those with high genomic instability, highlighting the therapeutic potential of TP in targeting malignant epithelial populations.

### 3.4 Pseudotime trajectory of TP target gene signatures in OC epithelial cells

**FIGURE 4:**
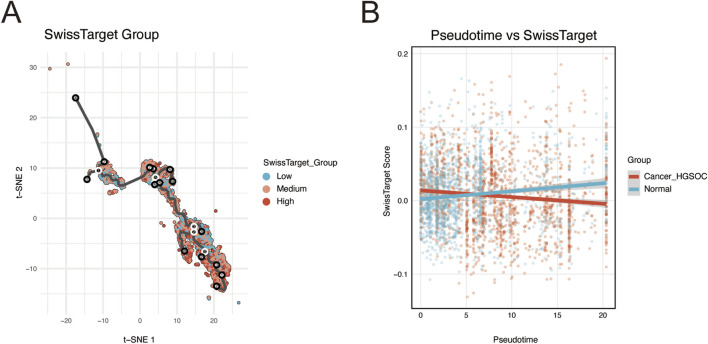
Relationship between the predicted TP target gene signature and cellular differentiation trajectories. **(A)** Pseudotime trajectory analysis projected onto a two-dimensional t-SNE space. The solid black line with numbered nodes delineates the inferred developmental lineage and distinct transitional states of the single cells. Individual cells are colored according to their SwissTarget score groups (Low, Medium, and High), demonstrating how the expression profile of predicted TP targets distributes along the developmental continuum. **(B)** Scatter plot detailing the dynamic changes of the SwissTarget gene score along the inferred pseudotime axis. Cells are categorized by sample origin into Cancer_HGSOC (red) and Normal (blue) groups. Linear regression trend lines with shaded confidence intervals illustrate distinct evolutionary patterns between the two cohorts: normal cells show a gradual increase in the SwissTarget score as pseudotime progresses, whereas cancer cells exhibit a near-horizontal regression line, indicating a negligible correlation with pseudotime and maintaining a relatively stable, divergent expression profile during cellular state transitions.

To further determine whether the expression of potential TP targets is associated with specific developmental stages or tumor progression states, we mapped the SwissTarget scores onto the constructed single-cell pseudotime trajectory. Visual inspection of the trajectory manifold revealed that cells with different TP target expression levels were not segregated into distinct developmental branches. Specifically, the High SwissTarget subpopulation (red) was ubiquitously dispersed throughout the trajectory, rather than being clustered at the root or terminal endpoints, exhibiting a distribution pattern similar to that of the Low and Medium groups (Figure 4A). Quantitative correlation analysis further corroborated this observation. In the Cancer_HGSOC group, the SwissTarget score demonstrated a negligible correlation with pseudotime, indicated by a near-horizontal regression line (Figure 4B). This suggests that the expression levels of TP targets remain relatively stable during the pseudo-temporal progression of OC. Collectively, these data indicate that the potential targets of TP are constitutively expressed across the tumor lineage and are not restricted to a specific differentiation state or “advanced stage” tumor population.

### 3.5 The high SwissTarget subpopulation cells exhibit unique SPP1-driven and matrix-dependent communication patterns

**FIGURE 5:**
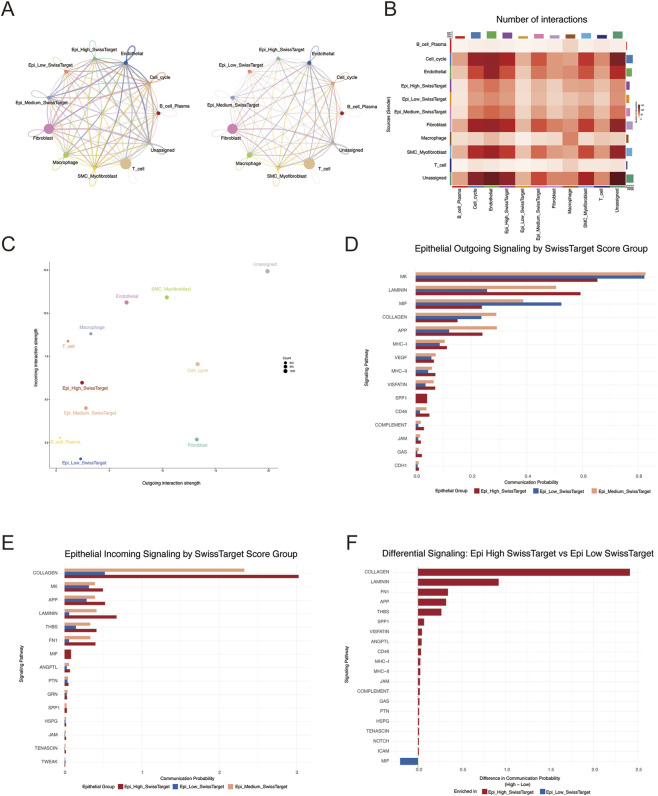
Intercellular communication landscape reveals distinct signaling networks associated with the predicted TP target gene signature. **(A)** Circle plots illustrating the global inferred intercellular communication networks among the identified cell populations. The nodes represent distinct cell types, and the connecting edges indicate signaling interactions. The line thickness is proportional to the overall interaction strength, visually highlighting the extensive crosstalk within the tumor microenvironment and showing how the Epithelial subpopulations (stratified into High, Medium, and Low SwissTarget score groups) integrate into this network. **(B)** Heatmap displaying the aggregate number of interactions between sender (rows) and receiver (columns) cell groups. The color gradient from light to dark red reflects the frequency of communication events. **(C)** Scatter plot characterizing the dominant signaling roles of various cell groups based on their overall outgoing (x-axis) and incoming (y-axis) interaction strengths. Dot size reflects the cell count. This analysis categorizes cell types into major senders (e.g., Fibroblasts) and major receivers (e.g., Endothelial cells, SMC_Myofibroblasts). Notably, the Epi_High_SwissTarget group exhibits elevated incoming and outgoing interaction strengths compared to the Epi_Low_SwissTarget group, indicating a more actively communicative state in the high-score tumor cells. **(D,E)** Bar plots comparing specific **(D)** outgoing and **(E)** incoming signaling pathway probabilities across the High (red), Medium (orange), and Low (blue) SwissTarget score Epithelial groups. The results demonstrate that High-score cells have significantly amplified communication probabilities, particularly functioning as major receivers for structural and oncogenic signals like COLLAGEN and LAMININ. **(F)** Bar plot quantifying the differentially enriched signaling pathways between the Epi_High and Epi_Low SwissTarget score groups. The x-axis represents the absolute difference in communication probability. Red bars indicate pathways significantly upregulated in the High-score group (such as COLLAGEN, LAMININ, FN1, and APP), while the blue bar represents pathways enriched in the Low-score group (such as MIF). This highlights a pronounced shift towards ECM remodeling and altered cellular adhesion signaling in cells with high TP target signatures.

To elucidate the interaction mechanisms between epithelial cells with high expression of potential TP targets and the TME, we employed “CellChat” to analyze intercellular communication. Comprehensive network analysis identified Fibroblasts and SMC_Myofibroblasts as the predominant signal sources within the TME, whereas Endothelial cells functioned as the primary signal receivers. Although the High, Medium, and Low SwissTarget epithelial subpopulations all exhibited extensive connectivity with the TME (Figures 5A,B), significant disparities were observed in their communication strengths and signaling preferences.

First, analysis of signaling roles revealed that while the outgoing interaction strengths were comparable across the three epithelial subgroups, the High SwissTarget group demonstrated a markedly enhanced incoming interaction strength, approximately four fold higher than that of the Low group (Figure 5C). This suggests that epithelial cells expressing high levels of TP targets exist in a state of heightened sensitivity to and dependence on environmental signals.

The High SwissTarget score group specifically dominated the transmission of the SPP1 (Osteopontin) signaling pathway and exhibited high activity in matrix remodeling signals, such as Laminin (Figures 5D,F). In contrast, the Low and Medium SwissTarget score groups preferentially secreted canonical growth factors, including MK (Midkine), MIF, and VEGF, potentially to maintain basal survival. Regarding incoming signaling, the High SwissTarget score group displayed a pronounced affinity for extracellular matrix (ECM) signals. Differential analysis indicated a significant enrichment of receptor-ligand interactions in the High SwissTarget score group, whereas these signals were negligible in the Low SwissTarget score group (Figures 5E,F). In conclusion, Figure 5 characterizes a unique “aggressive communication profile” for tumor cells with high expression of potential TP targets. These cells not only modulate the TME via SPP1 secretion but also reinforce their own matrix adhesion and survival capabilities through the high-intensity reception of ECM signals.

### 3.6 Identification of feature genes via integrated ML algorithms

**FIGURE 6:**
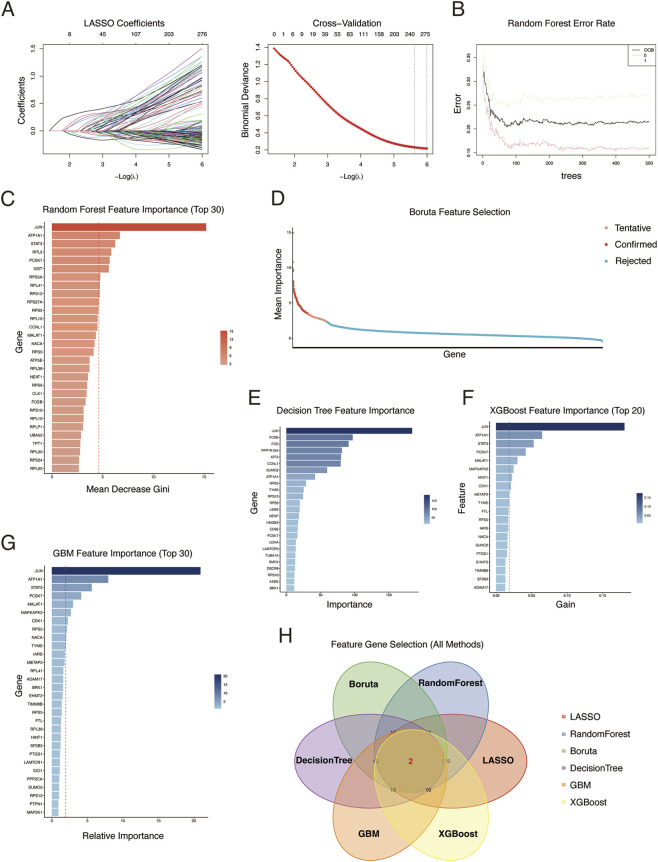
Screening of candidate therapeutic target using an integrated machine learning (ML) approach. **(A)** Feature selection using the LASSO regression model. The left panel shows the trajectory of independent variable coefficients against the L1-norm penalty term, illustrating the shrinkage of less important features to zero. The right panel displays the binomial deviance (cross-validation error) plotted against the -log(λ) sequence, with dotted lines indicating the optimal penalization parameter (λ) that minimizes the error rate for feature retention. **(B)** The OOB error rate of the Random Forest model plotted against the number of trees. The plot demonstrates the stabilization of the model’s error variance as the forest grows, validating the chosen model complexity. **(C)** The top 30 candidate features ranked by the Mean Decrease Gini index in the Random Forest analysis, highlighting genes (such as *JUN* and *ATP1A1*) that contribute most significantly to node purity and classification accuracy. **(D)** Feature importance assessment using the wrapper-based Boruta algorithm. Features are plotted by their mean importance (Z-score) and statistically classified into confirmed (red), tentative (orange), or rejected (blue) categories compared to randomized shadow features, effectively filtering out noise. **(E–G)** Bar plots illustrating the top-ranked feature genes derived from three additional models: **(E)** Decision Tree (ranked by overall importance), **(F)** XGBoost (ranked by Gain), and **(G)** GBM (ranked by relative importance). Notably, across these distinct algorithmic approaches, specific targets such as *JUN* consistently emerge with the highest predictive value. **(H)** Venn diagram illustrating the intersection of the candidate gene sets identified by all six distinct ML algorithms (Boruta, Random Forest, LASSO, Decision Tree, GBM, and XGBoost).

To screen for robust diagnostic markers between High-SwissTarget and Low-SwissTarget epithelial cells, we implemented a comprehensive workflow combining six ML algorithms. First, LASSO regression was utilized to minimize overfitting and eliminate redundant features. The optimal penalty parameter (λ) was determined via 10-fold cross-validation based on minimum binomial deviance, effectively narrowing the candidate gene set (Figure 6A).

Subsequently, we evaluated feature importance using ensemble and tree-based methods. The Random Forest (RF) model achieved error stability as the number of trees increased (Figure 6B), identifying *JUN* as the most significant gene based on the Mean Decrease Gini index (Figure 6C). To further ensure robustness, the Boruta algorithm was applied to categorize feature relevance into confirmed, tentative, and rejected attributes (Figure 6D). High concordance was observed across Decision Tree, XGBoost, and GBM models, all of which prioritized *JUN* as the top-ranking feature based on importance scores and gain values (Figures 6E–G).

Finally, to identify the most reliable biomarker, we performed an intersection analysis of the candidate genes derived from all six algorithms (LASSO, RF, Boruta, Decision Tree, GBM, and XGBoost). As illustrated in the Venn diagram, two optimal feature genes, *JUN* and *ATP1A1,* were consistently identified across all methods (Figure 6H; Supplementary Table S2).

### 3.7 Diagnostic performance and validation of identified feature genes

**FIGURE 7:**
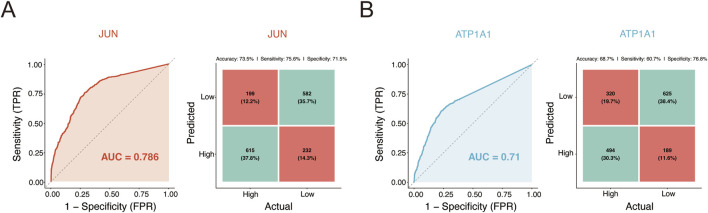
Diagnostic performance of the identified targets *JUN* and *ATP1A1*. **(A)** Evaluation of JUN as a predictive biomarker. The left panel shows the ROC curve, which plots Sensitivity (True Positive Rate) against 1 - Specificity (False Positive Rate). The AUC reaches 0.786, indicating a robust diagnostic capability. The right panel displays the corresponding confusion matrix, illustrating the model’s classification performance between predicted and actual groups (High vs. Low). For *JUN*, the model achieved an overall accuracy of 73.5%, successfully identifying true positives with a sensitivity of 75.6% and true negatives with a specificity of 71.5%. **(B)** Evaluation of *ATP1A1* as a predictive biomarker. The ROC curve (left) demonstrates an acceptable diagnostic value with an AUC of 0.71. The confusion matrix (right) details the classification outcomes, showing an overall accuracy of 68.7%. While *ATP1A1* exhibits a lower sensitivity (60.7%) compared to *JUN*, it demonstrates a slightly higher specificity (76.8%). Together, these plots validate that both *JUN* and *ATP1A1* possess significant and complementary diagnostic value for effectively stratifying the samples.

To further validate the reliability of the identified targets, we analyzed the predictive performance of *JUN* and *ATP1A1*. The classification performance of these genes were evaluated using ROC curve analysis and confusion matrices. *JUN* exhibited a robust discriminative ability with an AUC of 0.786 (95% CI: 0.764-0.808) (Figure 7A). The confusion matrix for *JUN* showed a prediction accuracy of 73.5%, with a sensitivity of 75.6% and a specificity of 71.5%. Similarly, *ATP1A1* also demonstrated good predictive potential, achieving an AUC of 0.71 (95% CI: 0.686-0.734) (Figure 7B), with an accuracy of 68.7% and a specificity of 76.8%. These results collectively suggest that *JUN*, and to a lesser extent *ATP1A1*, can serve as effective biomarkers for distinguishing OC, further supporting both these two genes as promising therapeutic targets for TP treatment.

Although both *JUN* and *ATP1A1* demonstrate potential as targets for TP, analysis of their biological functional hierarchy reveals that c-Jun, as a core component of the AP-1 transcription complex, acts as a master upstream regulator, whereas ATP1A1 functions primarily as a downstream effector. Furthermore, based on feature importance scores and gain values, all the mentioned ML algorithms consistently identified *JUN* as the top-ranked key feature gene. Consequently, this study prioritized the *JUN* gene for further investigation. However, we agree that the biological validation of *ATP1A1* constitutes a key direction for our future investigations.

To further evaluate the clinical relevance of *JUN* in OC, we analyzed the correlation between *JUN* expression and patient prognosis using the TCGA-OV cohort via Kaplan-Meier Plotter (Supplementary Figure S5). Survival analysis revealed that OC patients with high *JUN* expression exhibited significantly shorter Overall Survival (OS) compared to those with low expression (HR = 1.32, 95% CI: 1.12–1.55, log-rank p = 0.00087). Consistently, elevated *JUN* levels were also strongly associated with a decreased Progression-Free Survival (PFS) (HR = 1.31, 95% CI: 1.12–1.52, log-rank p = 0.00053). These macroscopic clinical data indicated that aberrant *JUN* overexpression is a critical risk factor associated with poor clinical outcomes and disease progression in OC.

### 3.8 TP directly engages and stabilizes c-Jun protein

To conclusively determine whether *JUN* serves as a direct pharmacological target of TP rather than merely a downstream functional mediator, we employed a combination of computational simulations and *in vitro* biophysical assays to evaluate molecular target engagement.

**FIGURE 8:**
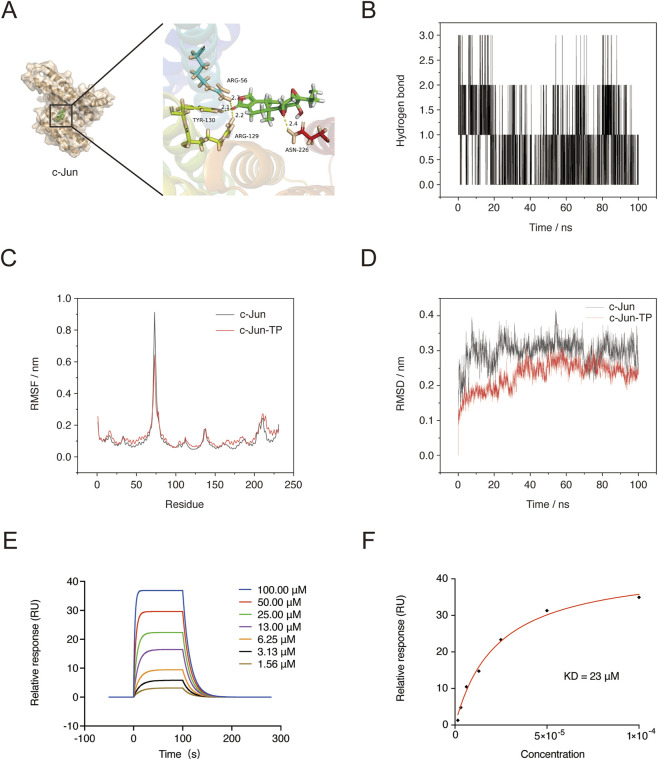
TP directly binds to and stabilizes c-Jun protein. **(A)** The predicted 3D binding mode of TP within the c-Jun protein pocket, highlighting specific hydrogen bond interactions with residues ARG-56, ARG-129, TYR-130, and ASN-226. Bond distances are indicated. **(B)** The number of intermolecular hydrogen bonds formed between TP and c-Jun monitored dynamically over the 100 ns molecular dynamics (MD) simulation at 310 K and 1.0 bar. **(C)** Root Mean Square Fluctuation (RMSF) analysis of individual residues in apo c-Jun (black) and the c-Jun-TP complex (red), evaluating local flexibility and structural compactness. **(D)** Root Mean Square Deviation (RMSD) trajectories of the backbone atoms in apo c-Jun (black) and the c-Jun-TP complex (red) over the 100 ns simulation time, demonstrating the stabilizing effect of TP binding on the overall protein conformation. **(E)** SPR sensorgrams characterizing the real-time binding kinetics between TP and immobilized c-Jun protein. TP was injected at an ascending concentration gradient ranging from 1.56 to 100.00 μM (1.56, 3.13, 6.25, 13.00, 25.00, 50.00, and 100.00 μM) at a flow rate of 20 μL/min, with an association time of 100 s and a dissociation time of 180 s. **(F)** Steady-state affinity fit curve derived from the SPR assay, calculating an equilibrium dissociation constant (KD) of 23 µM.

First, molecular docking was performed to delineate the binding mode between TP and the c-Jun protein. The simulated binding pose revealed that TP successfully occupies the active pocket of c-Jun, establishing critical hydrogen bonds with specific amino acid residues, including ARG-56, ARG-129, TYR-130, and ASN-226 (Figure 8A). Trajectory analysis across the 100 ns simulation confirmed the continuous maintenance of these hydrogen bonds, indicating a highly stable interaction interface (Figure 8B). This structural stabilization was further corroborated by Root Mean Square Fluctuation (RMSF) analysis, which showed restricted local flexibility in key residue regions upon TP binding (Figure 8C). Furthermore, Root Mean Square Deviation (RMSD) analysis demonstrated that the c-Jun-TP complex exhibited a notably lower and more stable trajectory compared to the apo c-Jun protein, suggesting that the binding of TP significantly stabilizes the overall conformation of the protein (Figure 8D). To experimentally validate these computational predictions and verify direct physical interaction *in vitro*, a Surface Plasmon Resonance (SPR) assay was conducted using purified human c-Jun protein. Varying concentrations of TP (ranging from 1.56 to 100.00 µM) were flowed over the c-Jun-immobilized sensor chip. The resulting sensorgrams displayed a classic, concentration-dependent binding response (Figure 8E). Steady-state affinity analysis yielded an equilibrium dissociation constant (KD) of 23 µM (Figure 8F). Collectively, these computational and biophysical data provide definitive, direct evidence that TP physically binds to and engages c-Jun, firmly establishing it as a direct pharmacological target.

Given that c-Jun maintains its own expression through a classical positive auto-regulatory transcriptional loop, we hypothesized that the direct structural binding of TP would impair c-Jun transactivation, thereby driving a secondary collapse of its intracellular expression and dismantling the chemoresistant phenotype.

### 3.9 Experimental validation of the effect of TP on CDDP resistance in OC

The development of platinum resistance is frequently accompanied by the aberrant expression of key resistance-related genes or transcription factors. To verify the acquired CDDP resistance of SKOV3/CDDP cell line, cell viability was evaluated following CDDP treatment for 48h. As shown in Supplementary Figure S2, CDDP inhibited the proliferation of both parental SKOV3 and resistant SKOV3/CDDP cells in a dose-dependent manner. The IC_50_ of CDDP was 1.880 μg/mL (95% CI: 1.678-2.102) in SKOV3 cells, whereas the IC_50_ markedly increased to 5.101 μg/mL (95% CI: 4.076-6.464) in SKOV3/CDDP cells, yielding a calculated resistance index (RI) of 2.71. These results demonstrate that SKOV3/CDDP cells exhibit a significant and stable resistance to CDDP compared with parental SKOV3 cells, thereby validating this model for the subsequent assays.

**FIGURE 9:**
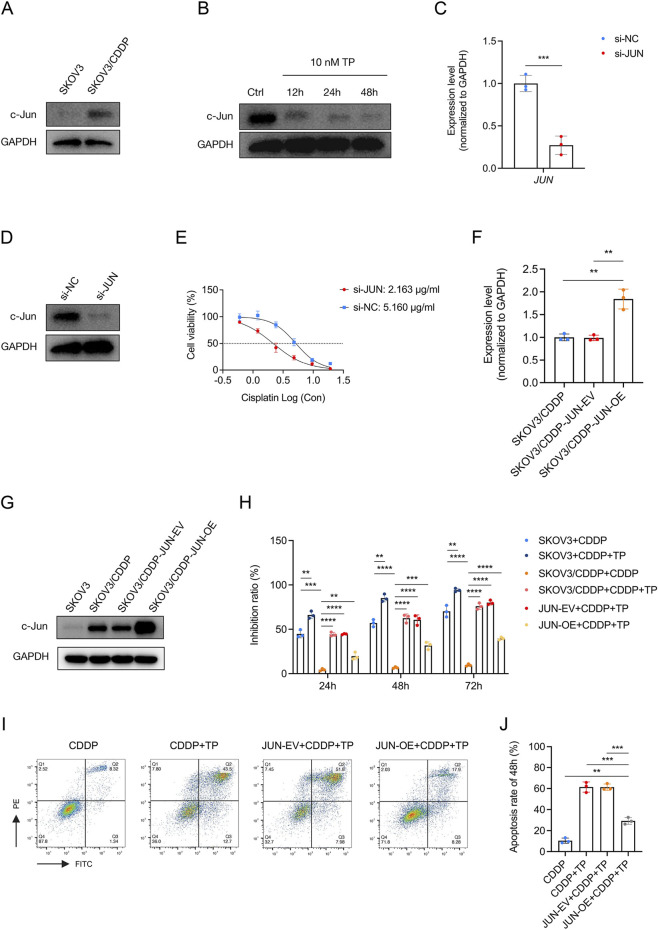
TP sensitizes SKOV3/CDDP cells to CDDP by regulating c-Jun expression. **(A)** Western blot analysis comparing basal c-Jun protein expression between parental SKOV3 and SKOV3/CDDP cells. The results demonstrate a significant upregulation of c-Jun in the SKOV3/CDDP cells, linking its expression to chemoresistance. **(B)** Time-dependent effect of TP on c-Jun expression. SKOV3/CDDP cells treated with 10 nM TP for 12, 24, and 48h show a progressive reduction in c-Jun protein levels, indicating that TP effectively suppresses c-Jun accumulation. **(C,D)** Validation of *JUN* knockdown. Relative *JUN* mRNA **(C)** and c-Jun protein **(D)** expression in SKOV3/CDDP cells transfected with si-JUN or si-NC, confirming successful gene silencing (p = 0.001 for mRNA). **(E)** Cell viability curves and corresponding IC_50_ values of SKOV3/CDDP cells treated with varying concentrations of CDDP for 48h. Silencing *JUN* markedly decreased the IC_50_ from 5.160 μg/mL to 2.163 μg/mL, demonstrating that reducing c-Jun expression partially restores CDDP sensitivity. **(F,G)** Validation of c-*Jun* overexpression. Relative *JUN* mRNA **(F)** and c-Jun protein **(G)** levels confirm the successful establishment of the *JUN* overexpressing cell line (SKOV3/CDDP-JUN-OE) compared to the empty vector control (SKOV3/CDDP-JUN-EV) (p = 0.0028 for mRNA) and SKOV3/CDDP cells (p = 0.0032 for mRNA). **(H)** Cell viability inhibition rates determined by CCK-8 assay for the indicated cell groups treated with CDDP or combination therapy (CDDP + TP) for 24, 48, and 72h. Co-treatment with TP significantly enhanced the proliferation inhibition of SKOV3/CDDP cells compared to CDDP monotherapy. Notably, the overexpression of c-Jun (JUN-OE+CDDP+TP group) significantly attenuated this TP-mediated sensitization, allowing cells to maintain their viability. Exact p-values for key comparisons are as follows: SKOV3+CDDP vs. SKOV3+CDDP+TP (24 h: p = 0.0033; 48 h: p = 0.0011; 72 h: p = 0.0037); SKOV3+CDDP vs. SKOV3/CDDP+CDDP (24 h: p = 0.0001; 48 h: p < 0.0001; 72 h: p < 0.0001); SKOV3/CDDP+CDDP vs. SKOV3/CDDP+CDDP+TP (24 h: p < 0.0001; 48 h: p < 0.0001; 72 h: p < 0.0001); SKOV3/CDDP+CDDP vs. JUN-EV+CDDP+TP (24 h: p < 0.0001; 48 h: p < 0.0001; 72 h: p < 0.0001); SKOV3/CDDP+CDDP vs. JUN-OE+CDDP+TP (24 h: p = 0.0035; 48 h: p = 0.0006; 72 h: p < 0.0001). **(I)** Representative flow cytometry plots (Annexin V/PI staining) analyzing cellular apoptosis in the indicated groups after 48h of treatment. **(J)** Quantitative analysis of the total apoptosis rates from **(I)**. The combination of CDDP + TP induced substantial apoptosis of SKOV3/CDDP cells compared to CDDP alone; however, this synergistic pro-apoptotic effect was significantly reversed by c-Jun overexpression, confirming that TP overcomes CDDP resistance primarily via the targeted downregulation of *JUN*. Exact p-values for key comparisons are:CDDP vs. CDDP+TP+JUN-OE, p = 0.0013, CDDP+TP vs. CDDP+TP+JUN-OE, p = 0.0007; CDDP+TP+JUN-EV vs. CDDP+TP+JUN-OE, p = 0.0002. Data are presented as the mean ± SD of three independent experiments (n = 3). *, p < 0.05; p < 0.01; ***, p < 0.001; ****, p < 0.0001.

Next, to investigate the potential role of the *JUN* gene in OC platinum resistance, we evaluated its expression in the parental SKOV3 cell line and the platinum-resistant SKOV3/CDDP cell line. Western blot analysis (Figure 9A and Supplementary Figure S6A) confirmed that the expression of c-Jun, the transcription factor encoded by JUN, was markedly higher in SKOV3/CDDP cells than in SKOV3 cells. These results suggest that the overexpression of c-Jun may be closely associated with the CDDP-resistant phenotype in SKOV3/CDDP cells.

Based on the aforementioned analysis, *JUN* emerges as a potential therapeutic target for TP in OC. We subsequently investigated whether TP could specifically modulate c-Jun expression in CDDP-resistant cells. As shown in Figure 9B and Supplementary Figure S6B, compared to the untreated control group, TP treatment induced a significant and progressive time-dependent decrease in intracellular c-Jun protein levels. Expression was substantially reduced at 12h and almost completely eradicated by 48h. These findings conclusively demonstrate that TP efficiently and persistently inhibits c-Jun protein expression in SKOV3/CDDP cells in a time-dependent manner, suggesting this may be a key molecular mechanism underlying the TP-mediated reversal of platinum resistance in OC.

To further elucidate the biological function of the *JUN* gene in OC platinum resistance, we performed an *in vitro* knockdown of *JUN* in SKOV3/CDDP cells using siRNA. qRT-PCR results (Figure 9C) showed a highly significant decrease of *JUN* in the si-JUN group compared to the si-NC group. Western blot analysis (Figure 9D) further verified that the protein expression of the transcription factor c-Jun was markedly suppressed in the si-JUN group. To demonstrate the driving role of aberrantly overexpressed *JUN* in maintaining platinum resistance, we assessed the viability of SKOV3/CDDP cells treated with varying concentrations of CDDP 48h post-transfection with si-NC or si-JUN using a CCK-8 assay. As depicted in Figure 9E, compared to the si-NC group, cells transfected with si-JUN showed a profound restoration of sensitivity to CDDP. Specifically, the si-NC group, which maintained high *JUN* expression, exhibited an IC_50_ value of 5.160 μg/mL (95% CI: 4.628-5.767) for CDDP, displaying a typical highly resistant phenotype. In contrast, effective knockdown of *JUN* in the si-JUN group reduced the IC_50_ value to 2.163 μg/mL (95% CI: 1.971-2.375), resulting in a significant leftward shift of the cell survival curve. This result intuitively and strongly demonstrates that the *JUN* gene plays a pivotal role in the progression of OC platinum resistance. Specific blockade of the c-Jun signaling pathway significantly reversed the CDDP-resistant phenotype in PROC, validating the potential of *JUN* as a crucial therapeutic target.

To delve deeper into the core mechanism by which *JUN* maintains the platinum-resistant phenotype, we established a JUN-overexpressing SKOV3/CDDP cell line (SKOV3/CDDP-JUN-OE) and a negative control cell line transfected with an empty vector (SKOV3/CDDP-JUN-EV). As shown in Figures 9F,G, compared to untreated SKOV3/CDDP cells and the SKOV3/CDDP-JUN-EV group, the relative *JUN* mRNA level in the SKOV3/CDDP-JUN-OE group was remarkably upregulated. Concurrently, western blot results confirmed a distinct increase in c-Jun protein expression in the SKOV3/CDDP-JUN-OE group. No significant difference in the expression of *JUN* was observed between the blank and empty vector control groups, excluding any non-specific effects of the transfection procedure.

Cell viability was then quantitatively analyzed using the CCK-8 assay at 24, 48, and 72h post-treatment across different experimental groups. As illustrated in Figure 9H, treatment with CDDP alone yielded a profoundly lower inhibition ratio in the SKOV3/CDDP cells compared to the parental SKOV3 cells, confirming the resistant phenotype. In contrast, combined treatment with TP (CDDP+TP group) significantly inhibited proliferation, exhibiting a clear time-dependent increase in the inhibition rate and reaffirming that TP acts synergistically with CDDP to induce cytotoxicity. The growth inhibition trend in the JUN-EV +CDDP+TP group was consistent with that of the SKOV3/CDDP+CDDP+TP group, ruling out plasmid interference. Notably, the cytotoxicity exerted by the TP and CDDP combination was significantly counteracted following high *JUN* gene expression. Compared to the JUN-EV+CDDP+TP group, the proliferation inhibition rates in the JUN-OE+CDDP+TP group decreased substantially at 24, 48, and 72h, demonstrating distinct resistance to the combination therapy. These results strongly indicate that maintaining high intracellular levels of *JUN* effectively antagonizes the synergistic cytotoxic effects of TP and CDDP.

To further verify that *JUN* is a key target for TP in reversing OC platinum resistance, cell apoptosis was evaluated using flow cytometry. As presented in Figures 9I,J, the total apoptosis rate in the CDDP monotherapy group was only 10.44% (95% CI: 4.31-16.57). However, combination with TP (CDDP+TP group) induced massive cell apoptosis, characterized by a sharp increase in early and late apoptotic populations, surging to approximately 61.4% (95% CI: 49.38-73.47). This indicates that TP promotes CDDP-induced lethal apoptosis in resistant cells. The apoptosis rate in the JUN-EV+CDDP+TP group showed no statistical difference compared to the CDDP+TP group. Following *JUN* overexpression (JUN-OE+CDDP+TP group), the high level of apoptosis triggered by the combination therapy was significantly reversed, with the total apoptosis rate being significantly reduced compared to the JUN-EV+CDDP+TP control. These data provide compelling evidence that high expression of *JUN* effectively resists the apoptotic program triggered by the synergistic action of TP and CDDP.

In conclusion, TP can re-sensitize SKOV3/CDDP cells to CDDP by targeting and suppressing the aberrant overexpression of *JUN*, thereby reversing platinum resistance in PROC cells.

## 4 Discussion

This study integrated scRNA-seq data with ML algorithms to elucidate the molecular mechanisms by which TP reverses platinum resistance in OC. By leveraging scRNA-seq, we precisely mapped the pharmacological targets of TP in a highly genomically unstable and aggressive epithelial cell subpopulation, effectively eliminating background interference from non-malignant stromal components. Furthermore, the application of ML algorithms transformed this high-dimensional screening process into a streamlined and efficient workflow. This synergistic strategy surpasses traditional screening methods in both accuracy and efficiency, offering a novel paradigm for deciphering the complex pharmacological mechanisms of active compounds derived from traditional Chinese medicine.

Bioinformatic analysis of the scRNA-seq data revealed that malignant epithelial cells exhibiting high expression of TP targets possess distinct, aggressive intercellular communication features. This subpopulation demonstrates a profound dependence on ECM signals, particularly laminin and collagen. These interactions activate integrin-mediated survival signaling pathways within the cells, conferring resistance to anoikis during peritoneal dissemination (Buchheit et al., 2014; Lengyel, 2010). Additionally, these malignant epithelial cells dominate the secretion of osteopontin (SPP1), a pivotal cytokine known to drive tumor metastasis and chemoresistance (Shevde and Samant, 2014; Qian et al., 2021).

To further pinpoint the critical therapeutic targets of TP in OC, an ensemble of ML algorithms, including LASSO, Random Forest, Boruta, Decision Tree, XGBoost, and GBM, was employed to analyze the high-dimensional scRNA-seq data. This approach identified *JUN* and *ATP1A1* as core targets for TP. We prioritized the *JUN* gene for subsequent experimental validation because it not only exhibited exceptionally high feature weights across all algorithms but is also well-documented for its crucial role in driving tumor platinum resistance.

The *JUN* proto-oncogene encodes the c-Jun protein, a core subunit of the Activator Protein-1 (AP-1) transcription factor complex. As a ubiquitously expressed transcription factor, c-Jun acts as a central signal-transducing hub regulating cell proliferation, differentiation, and apoptosis (Shaulian and Karin, 2002). In various solid tumors, *JUN* is frequently aberrantly overexpressed, promoting cell proliferation by upregulating cyclins and driving epithelial-mesenchymal transition (EMT) via the upregulation of matrix metalloproteinases (Eferl and Wagner, 2003; Bejjani et al., 2019). Concurrently, under the selective pressure of platinum-based agents, the sustained activation of *JUN* upregulates anti-apoptotic proteins (e.g., Bcl-2, Bcl-xL) and enhances DNA repair capacities (Galluzzi et al., 2012). This *JUN*-mediated stress adaptation is a fundamental molecular mechanism underlying platinum resistance in tumor cells (Siddik, 2003).

Building upon these theoretical foundations, we implemented rigorous computational simulations and biophysical binding assays. MD simulations revealed that TP stably anchors within the active pocket of the c-Jun protein, effectively restricting its structural flexibility. Most importantly, SPR assay provided definitive physical evidence of direct target engagement, demonstrating that TP strongly binds to purified c-Jun protein with an equilibrium dissociation constant of 23 µM. These findings solidly establish c-Jun as a direct pharmacological target of TP.

This direct protein-level engagement elegantly elucidates the transcriptomic trends initially observed in the scRNA-seq analysis. It is well-documented that the c-Jun protein, acting as the core of the AP-1 complex, robustly activates its own transcription via a positive auto-regulatory loop by binding to the promoter region of the *JUN* gene (Shaulian and Karin, 2002). Our findings suggest that the direct physical binding of TP to the c-Jun protein impairs its structural integrity and functional transactivation capacity. Consequently, this target engagement collapses the AP-1 auto-regulatory transcriptional loop, fundamentally starving the cell of nascent *JUN* mRNA. This mechanism perfectly bridges the gap between the direct pharmacological engagement of the c-Jun protein and the profound suppression of JUN expression we observed *in vitro*.

Building upon these analytical findings and theoretical foundations, *in vitro* experiments confirmed that *JUN* is highly expressed in the PROC cell line SKOV3/CDDP, and targeted knockdown of *JUN* significantly restored the cellular responsiveness to CDDP. Crucially, TP efficiently and persistently suppressed the expression of *JUN* in SKOV3/CDDP cells in a time-dependent manner. Conversely, overexpression of *JUN* in SKOV3/CDDP cells significantly antagonized the sensitizing effects of TP. These results validate that *JUN* is the core therapeutic target for TP-mediated reversal of OC platinum resistance. By specifically downregulating *JUN* expression, TP dismantles the pro-survival network of PROC cells, thereby effectively re-sensitizing them to CDDP.

Furthermore, previous studies have established that SPP1 is a canonical transcriptional target of c-Jun (El-Tanani et al., 2006). This mechanistic link elucidates the communication features observed in our bioinformatic analysis: TP likely reverses OC platinum resistance not only by downregulating *JUN* to sever intracellular survival pathways (Chugh et al., 2012) but also by subsequently inhibiting the secretion of SPP1 into the TME. This effectively disrupts the pro-tumorigenic crosstalk between malignant cells and the TME (Liu et al., 2025; Wei et al., 2019). While this hypothesis enriches the molecular framework of TP, it warrants further experimental validation.

CDDP resistance in OC is widely recognized as a highly complex, multifactorial phenomenon, driven by multiple mechanisms, including altered intracellular drug accumulation, enhanced DNA damage repair capacity, and the hyperactivation of specific pro-survival signaling pathways. Recognizing that extensive prior research has widely profiled the reverse effect of TP on CDDP resistance in OC (Li et al., 2022; Zhong et al., 2021; Le et al., 2021), our current study deliberately focused on elucidating the deeper, upstream molecular mechanism of how TP reverses the CDDP-resistance of OC by combining scRNA-seq databases and ML analysis. Specifically, we identified the aberrant overexpression of the *JUN* gene as a critical component of the resistant phenotype in SKOV3/CDDP cells. Nevertheless, given the highly interconnected nature of chemoresistance networks, it is plausible that the *JUN* pathway intersects with the classical resistance mechanisms mentioned above. Therefore, while our findings highlight the primary role of *JUN*, future comprehensive investigations are warranted to explore whether TP-mediated regulation of c-Jun concomitantly modulates the expression of chemoresistance-related protein markers, such as specific ABC efflux transporters. Such studies will further fully elucidate the precise, multifaceted mechanisms by which TP overcomes CDDP resistance in OC.

Despite the promising findings above, several limitations in the current study must be acknowledged. Computationally, our scRNA-seq target screening relied on primary, untreated HGSOC datasets rather than clinically validated, pre-treated PROC samples. Furthermore, treating single cells as independent observations may introduce pseudoreplication, and the CellChat-derived intercellular communications remain transcriptomic inferences. Future studies employing pseudobulk aggregation and functional co-culture assays are required to substantiate these initial predictions. Experimentally, our *in vitro* functional validations were confined to a single resistant cell line (SKOV3/CDDP) with empirically determined sample sizes. Validating these mechanisms across a broader panel of resistant cell lines and patient-derived organoids (PDOs), guided by strict *a priori* power analyses, will be crucial to ensure generalizability. Most importantly, the absence of *in vivo* validation limits the immediate translational impact of our findings, as cellular models cannot fully recapitulate the complex tumor microenvironment, systemic pharmacokinetics, and potential off-target toxicities. Therefore, future comprehensive investigations incorporating matched pre- and post-chemotherapy clinical cohorts, robust *in vivo* models (such as PDX models) integrated with targeted delivery systems, and rigorous clinical trials are imperative to definitively establish the therapeutic safety and clinical efficacy of targeting c-Jun with TP in PROC.

In summary, by combining scRNA-seq data and ML, this study successfully identified and validated c-Jun as the target for TP in overcoming OC platinum resistance. This target screening strategy overcomes the inherent limitations of traditional bulk transcriptomics, offering new insights into the anti-tumor mechanisms of botanical monomers. Ultimately, these findings establish a robust theoretical foundation for the future development of TP-loaded nanomedicines tailored for the precise treatment of PROC.

## Data Availability

The original contributions presented in the study are included in the article/Supplementary Material, further inquiries can be directed to the corresponding authors.
