# Supplementary figures and images for: Triptolide targets JUN to reverse cisplatin resistance of ovarian cancer: insights from single-cell transcriptome analysis and machine learning validation
Source: Front Pharmacol. 2026 Jul 9;17:1850438. doi: 10.3389/fphar.2026.1850438 (PMC13391933; doi:10.3389/fphar.2026.1850438)

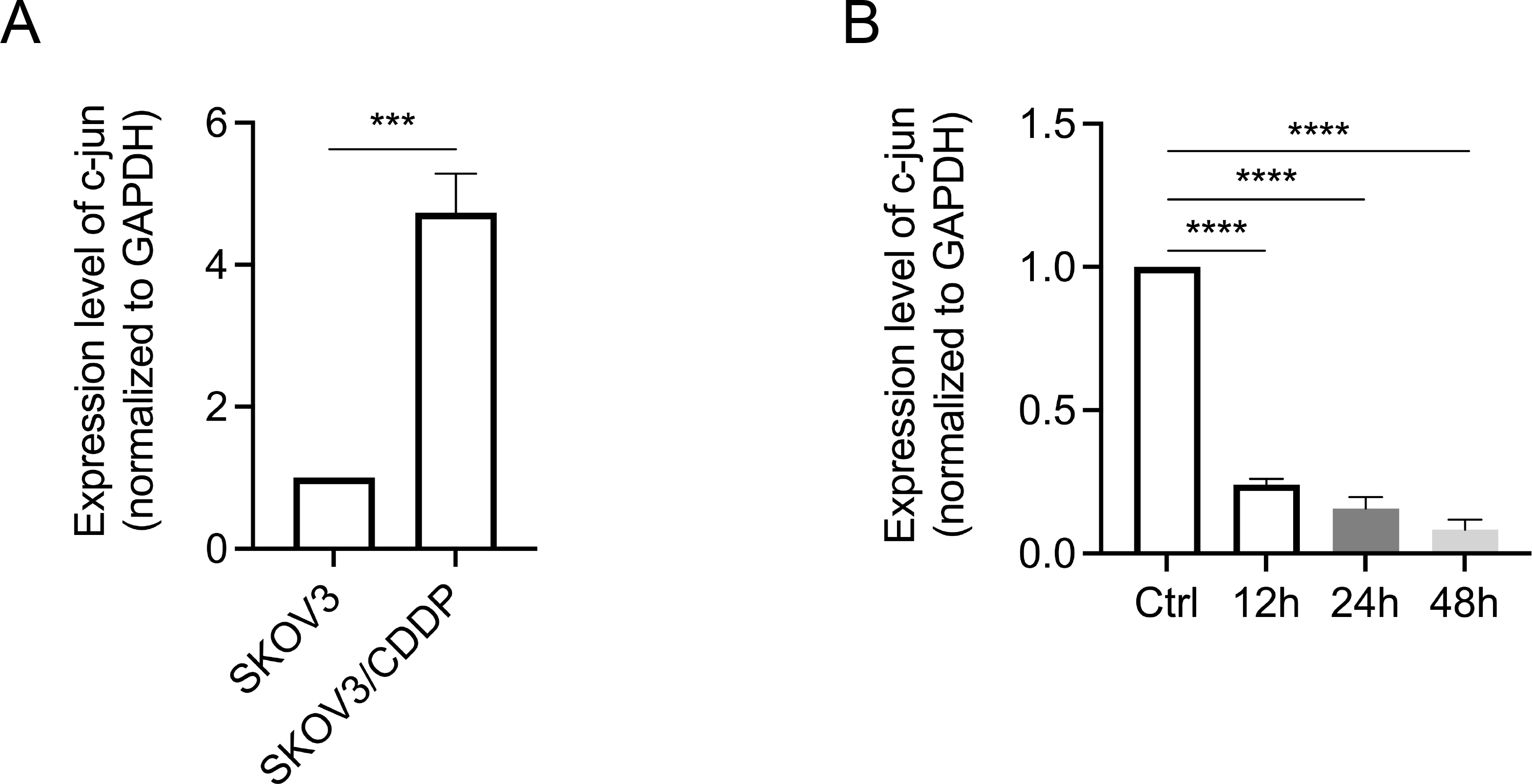

Supplement: Supplementary file 1 [file Image6.tif]

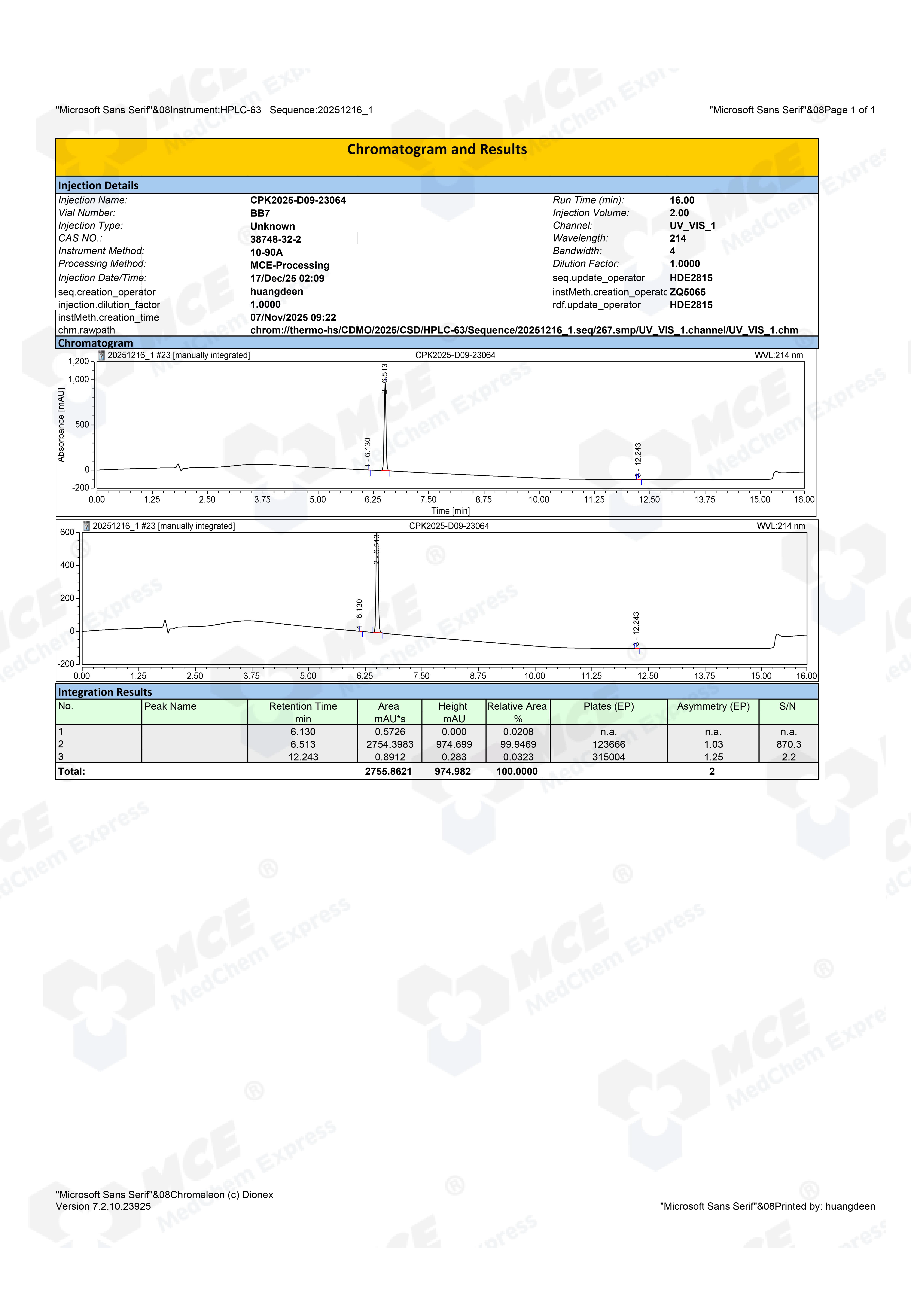

Supplement: Supplementary file 2 [file Image3.tif]

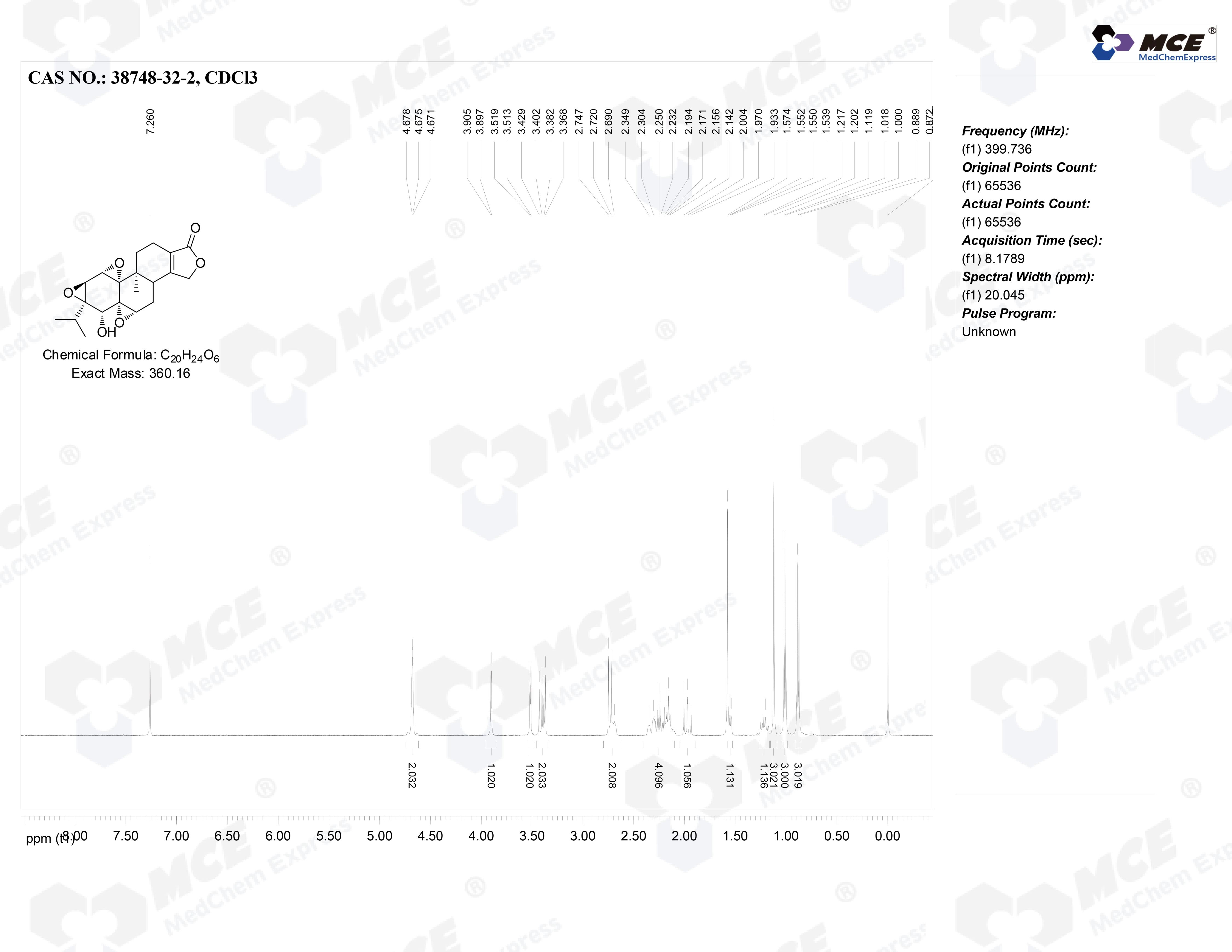

Supplement: Supplementary file 3 [file Image4.tif]

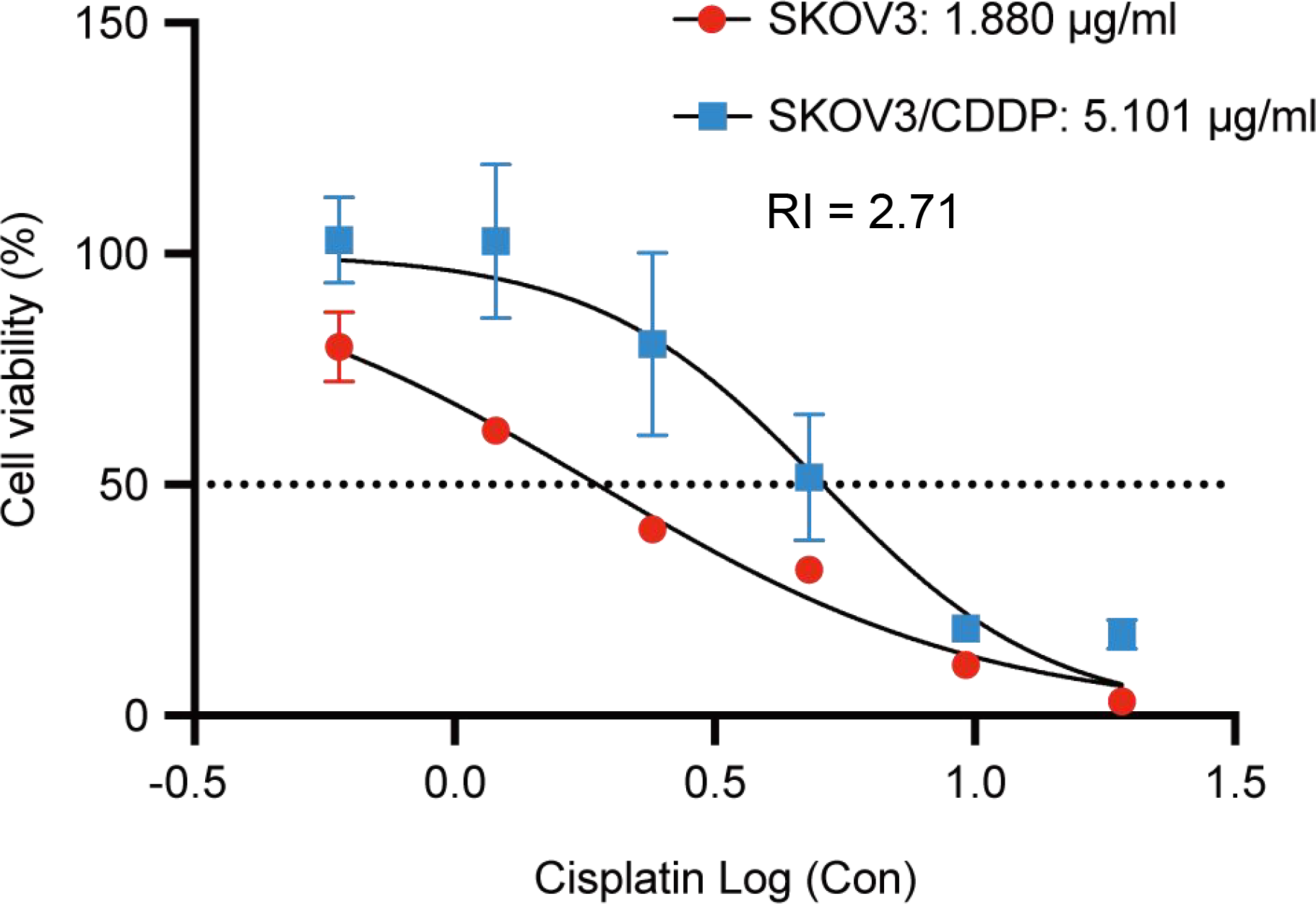

Supplement: Supplementary file 4 [file Image2.tif]

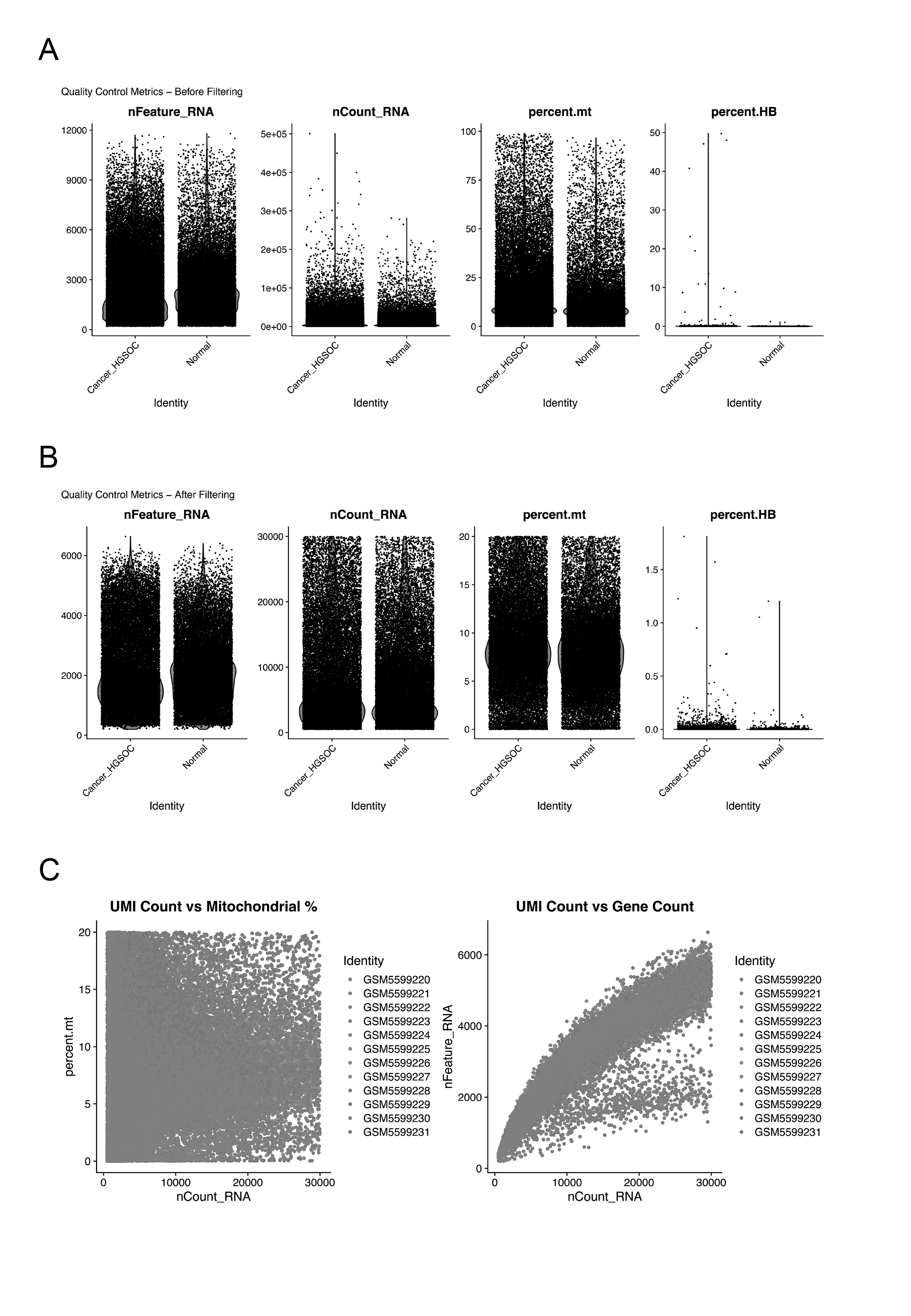

Supplement: Supplementary file 5 [file Image1.tif]

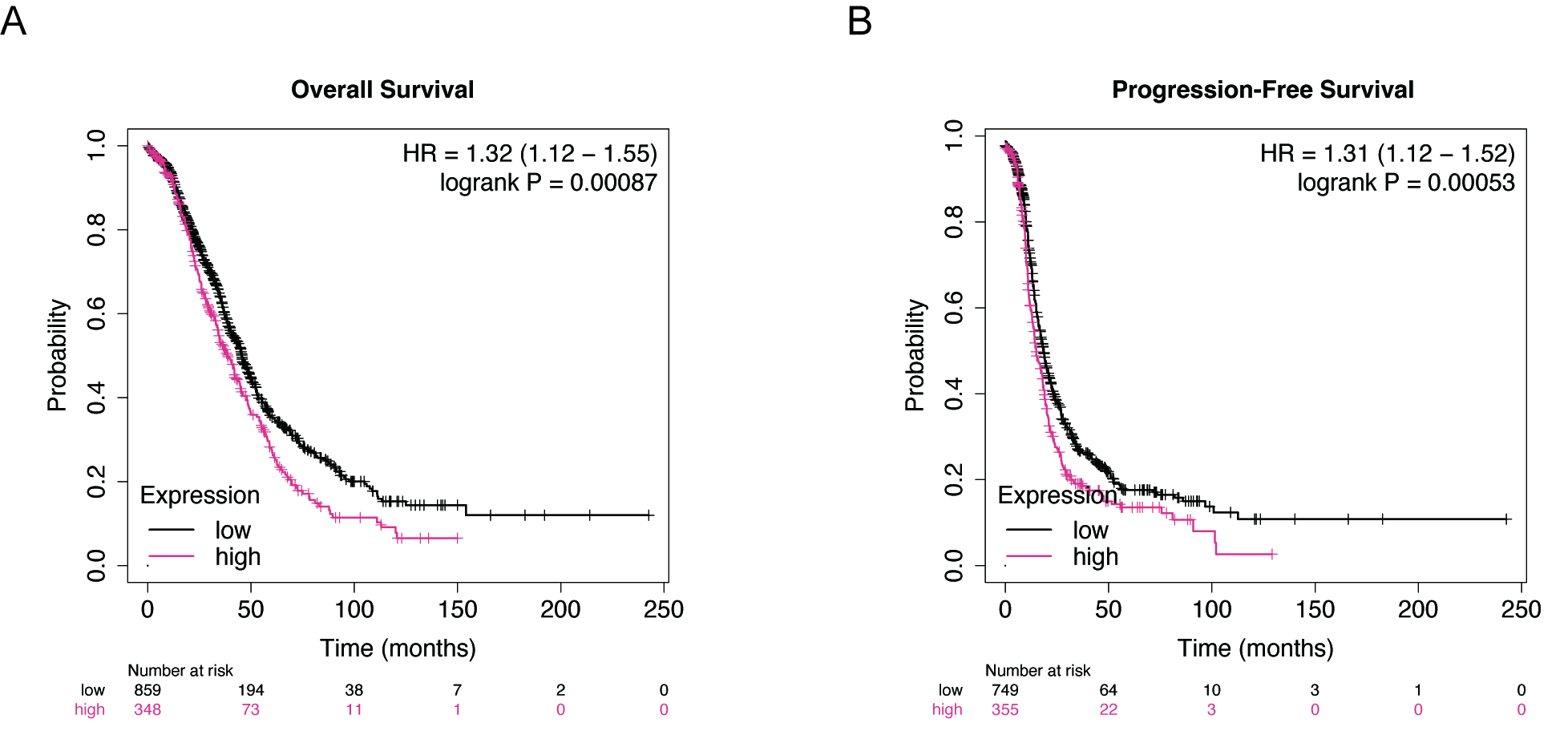

Supplement: Supplementary file 6 [file Image5.tif]
